# Cyber Victimization Is Associated With Eating Disorder Psychopathology in Adolescents

**DOI:** 10.3389/fpsyg.2018.00987

**Published:** 2018-06-14

**Authors:** Jose H. Marco, M. Pilar Tormo-Irun

**Affiliations:** ^1^Facultad de Psicología, Magisterio y Ciencias de la Educación, Universidad Católica de Valencia San Vicente Mártir, Valencia, Spain; ^2^Universidad Internacional de Valencia (VIU), Valencia, Spain

**Keywords:** cyber bullying, cyber victimization, eating disorders psychopathology, diet, body dissatisfaction, overweight preoccupation

## Abstract

**Introduction:** Technology is constantly evolving in a vast number of fields. In this way, cyber victimization is associated with psychopathology, and body appearance is a primary target of cyberbullies. Thus, the literature demonstrates a clear association between bullying and unhealthy eating behaviors in adolescents. However, studies that have examined the association between cyber victimization and eating disorder psychopathology are scarce.

**Objective:** (1) To analyze whether there are differences in the cyber victimization scores depending on the gender, controlling for age; (2) to analyze whether cyber victimization is negatively associated with eating disorder psychopathology; (3) to analyze whether this association is moderated by the level of Appearance Evaluation.

**Method:** Participants in the present study included 676 adolescents, 367 girls and 309 boys from several cities of Spain. The mean age for the overall sample was 14.28 years (*SD* = 1.65), ranging from 12 to 19. The participants filled out the questionnaires ECIPQ, MBSRQ, and EAT.

**Results:** Cyber victimization was associated with eating disorders psychopathology, Appearance Evaluation, and Overweight Preoccupation. Appearance Evaluation moderated the association between Cyber victimization and the eating disorder psychopathology.

**Conclusion:** It is necessary to assess vulnerability to eating disorders in adolescents who have been victims of cyberbullying.

## Introduction

Cyberbullying is a widespread phenomenon among adolescents, and frequency rates range between 6.6% and 44.1% (Kowalski and Limber, 2007; Calvete et al., 2010; Garaigordobil, 2011; Patchin and Hinduja, 2013; Kowalski et al., 2014; Rice et al., 2015). Cyberbullying refers to a specific form of unwarranted, intentional, and prolonged aggression that appears between peers and occurs in the digital environment through the use of electronic media (Tokunaga, 2010).

Cyber victimization is associated with negative emotions such as anxiety, depression, and suicide ideation (Raskauskas and Stoltz, 2007; Hinduja and Patchin, 2010), low self-esteem (Beran and Li, 2007), difficulties in relationships due to developing an attitude of isolation and loneliness (Ybarra and Mitchell, 2004), school absenteeism (Hinduja and Patchin, 2008), and substance abuse (Selkie et al., 2015; Calvete et al., 2016).

Body appearance is a primary target of cyberbullies (Menzel et al., 2010) (e.g., receiving harassing emails or text messages about one's body, telling other children not to be friends with someone because s/he is fat, or being the target of derogatory jokes or sending derogatory images about his/her body over the Internet). Thus, Cassidy et al. (2009) found that over 30% of adolescents reported being cyberbullied due to their size or weight. In the same way, Mishna et al. (2010) found that 10% of adolescents reported that they were bullied online because of their appearance. Another study (Frisén et al., 2014) found that cyber victims reported a worse view of their general appearance and weight than non-cyber victims, and in studies with adolescents, Ramos-Salazar (2017) found that being cyberbullied by peers is associated with body dissatisfaction.

Thus, the literature demonstrates a clear association between bullying and unhealthy eating behaviors in adolescents (Menzel et al., 2010). Several studies found that weight-based negative interactions involving bullying, teasing, negative verbal commentary, or other forms of victimization were significant predictors of difficulties in regulating eating behavior, binge eating symptoms, and weight (Kaltiala-Heino et al., 2003; Sweetingham and Waller, 2008; Arat, 2015; Carmona-Torres et al., 2015; Copeland et al., 2015; Lee et al., 2017). However, studies examining the association between cyber victimization and adolescents' eating disorder psychopathology are scarce. Only the Ramos-Salazar (2017) study was carried out in non-clinical samples of adolescents. The results showed that cyber victimization experiences have a positive effect on dieting to lose weight. However, a limitation of this study is that it was carried out without specifically assessing the psychopathology of eating disorders. Ramos-Salazar (2017) only assessed eating behaviors with a single item about “Dieting to lose weight.”

In order to analyze whether cyber victimization is associated with eating disorder psychopathology, we should assess cyberbullied participants with standardized questionnaires that specifically assess eating disorder psychopathology. Moreover, the association between cyber victimization and eating disorder psychopathology could depend on other etiological variables of eating disorders. For example, if cyber victimization is associated with unhealthy behaviors toward food, this association could be moderated by other variables strongly related to the psychopathology of eating disorders, such as body dissatisfaction (Pennesi and Wade, 2016). Therefore, the question of whether cyber victimization is associated with the psychopathology of eating disorders in adolescents has not yet been answered.

Meta-analytic studies about the relationship between weight-based teasing and body dissatisfaction or eating disorder psychopathology suggested that this association was mediated by the age and gender of the participants (Menzel et al., 2010). However, studies about the consequences of cyber victimization based on gender are contradictory. Brown et al. (2014) found no differences in cyber victimization depending on gender. However, Frisén et al. (2014) found that the adolescents believed that cyberbullying was directed at the victims' appearance, especially when girls were cyber victims. In the same way, being a victim of appearance teasing seems to have a more profound effect on girls' body esteem than on boys' (Lunde et al., 2007). In addition, results from studies about the influence of the participants' age on cyber victimization are not consistent either. For example, previous studies found that cyber victimization was more common among 12–15 year olds (Tokunaga, 2010). However, other studies found that it was more common in young adolescents than in older adolescents (Frisén et al., 2014), whereas Brown et al. (2014) found no differences in age in the cyber victimization experience in adolescents. Thus, more research is needed to understand whether gender and age differences are important cyberbullying variables.

In order to extend the knowledge in this area of research, it would be necessary to confirm the associations between cyber victimization and eating disorder psychopathology and body dissatisfaction in adolescents.

The aims of the present study are: (1) To analyze whether there are differences in the cyber victimization scores depending on the gender, controlling for age; (2) to analyze whether cyber victimization is negatively associated with Appearance Evaluation, and positively associated with Overweight Preoccupation and eating disorder psychopathology; (3) to analyze whether Cyber victimization is associated with eating disorder psychopathology, with age, gender, and Overweight Preoccupation controlled, and whether this association is moderated by the adolescents' level of Appearance Evaluation.

## Methods

### Participants

Participants in the present study included 676 adolescents, 367 girls and 309 boys from several cities of Spain. The mean age for the overall sample was 14.28 years (*SD* = 1.65), ranging from 12 to 19. Participation was voluntary and anonymous, and participants did not receive any compensation for participating in the research. The inclusion criterion was that they had to be male or female adolescents between 12 and 19 years old, with informed consent signed by students and parents. We followed the World Health Organization (2003) definition of adolescents as people from 10 to 19 years old. The exclusion criterion was refusal to participate in the study, by either the students or the parents or both. Participants were given appropriate instructions to complete the protocol.

### Assessments and measures

#### Multidimensional body-self relations questionnaire-appearance scales; (MBSRQ-AS; Cash, 2000)

The MBSRQ-AS is a 34-item self-report inventory composed of five subscales with good psychometric properties in males and females: Appearance Evaluation, Appearance Orientation, Body Areas Satisfaction, Overweight Preoccupation, and Self-Classified Weight. For this study, we used two subscales: (a) the Appearance Evaluation subscale, composed of 7 items, which assesses feelings of satisfaction or dissatisfaction with one's appearance. Each item is scored on a 5-point scale and evaluates agreement (from 1: “Definitely disagree” to 5: “Definitely agree”). Higher scores indicate greater feelings of satisfaction with the figure and body; and (b) the Overweight Preoccupation subscale, composed of four items, which assesses fat anxiety, weight vigilance, dieting and eating restraint. Each item is scored on a 5-point scale and evaluates agreement (from 1: “Strongly disagree” to 5: “Strongly agree”), except the item about the frequency of diets (from 1: “Never” to 5: “Quite often”). Higher scores indicate greater preoccupation about weight and overweight. The score on each subscale consists of the average of the scores on its items (range 1–5). We utilized the Spanish version of the MBSRQ-AS (Marco et al., 2017). The scores showed good internal consistency and reliability in the present sample for Appearance Orientation (α = 0.93) and Overweight Preoccupation (α = 0.89).

#### Eating attitudes test (EAT-40; Garner and Garfinkel, 1979)

The Spanish version of the EAT-40 has 40 items answered on a 6–point Likert scale (Castro et al., 1991). It evaluates the main psychopathology of eating disorders, negative attitudes and fears, dysfunctional beliefs about food, and unhealthy behaviors associated with eating disorders, such as purging, binge-eating, and dieting. Each item is scored on a 6-point scale and evaluates frequency (from 0: “Always” to 3: “Rarely”). Higher scores indicate greater ED symptom severity (beliefs, behaviors and feelings). The score on the EAT consists of the sum of the scores on its items (range 0 to 120). In the Spanish version, the authors suggest that scores greater than a cut-off point of 21 indicate the possible presence of an ED (Castro et al., 1991; de Irala et al., 2008). The scores showed good reliability in the present sample (range α = 0.90).

#### European cyberbullying intervention project questionnaire (ECIPQ; Del Rey et al., 2015)

We used the Spanish version of the ECIPQ, composed of a 22-item Likert-type scale with five response options for frequency (from never = 0 to more than once a week = 3). The ECIPQ has two subscales (cyber victimization and cyber aggression). For our research, we only used the Cyber Victimization subscale, composed of 11 items, which assesses whether the participant has been a victim of specific behaviors such as insults, threats, taking personal information, identity theft, publishing information, intimate photos, retouched photos, and spreading rumors through messages, the Internet, and social networks in the past 2 months. Higher scores indicate greater frequency. The score on the Cyber Victimization subscale consists of the sum of the scores on its items (range 0–33). In this study, we consider that the participant has been a victim of cyberbullying if s/he has experienced at least one of the behaviors mentioned above in the past 2 months. The Spanish version offers adequate reliability (α = 0.83), and in our sample it showed excellent internal consistency (α = 0.87).

### Procedure

First, we contacted 20 schools in several cities in Spain by telephone to explain the aim of the study. We asked if they were interested in collaborating in this research, and 12 (60%) schools decided to participate. In each school, a lecture was given to the parents of the students about the consequences of cyberbullying, and then their informed consent was requested for their children's participation in the investigation. The students provided written agreement, and the parents gave written consent for participation in the study. The students filled out the questionnaires during their normal school day at the beginning of the day (9 h). This study received the ethical approval of the University Ethics Committees.

To reduce social desirability, the participants were assured that the answers would be anonymous, and the order of presentation of the instruments was counter-balanced. The questionnaires selected for this research (EAT-40, MBSRQ-AS, ECIPQ) are designed for use in school contexts, and they are commonly used with high-risk populations, showing great reliability and validity in numerous studies (e.g., Marco et al., 2017; Calvete et al., 2018). Standardized test administration procedures were followed for all measures.

### Statistical procedure

First, a univariate analysis of variance (ANOVA) was carried out to compare the participating girls and boys on their Cyber victimization scores, with Age as covariate. Second we performed zero-order correlations for the variables. Third, we performed one hierarchical regression analysis. We used Appearance Evaluation and Cyber victimization as predictor variables, and the eating disorders psychopathology (EAT) as the dependent variable. In the first step, Age, Gender, and Overweight Preoccupation were entered. In the second step, Cyber victimization was entered. In the third step, Appearance Evaluation was entered. In the fourth step, the interaction term between Cyber victimization and Appearance Evaluation was entered. In each step, centered variables were used to avoid multicollinearity (Frazier et al., 2004). If the addition of the interaction term in the fourth step added significant predictive variance to the regression model, this indicated a moderating effect of Appearance Evaluation in the association between Cyber victimization and the eating disorder psychopathology (EAT) (Cohen and Cohen, 1983; Frazier et al., 2004). Analyses were performed using the enter method. Data were analyzed using SPSS 23 (SPSS, Chicago, IL).

## Results

We found that 57.5%, *n* = 389, of participants were victims of insults, threats, taking personal information, identity theft, publishing information, intimate photos, retouched photos, and spreading rumors through messages, the Internet, and social networks in the past 2 months.

The results indicated that there were no statistically significant differences between girls (*M* = 2.97, *SD* = 5.72) and boys on the Cyber victimization score [*M* = 3.11, *SD* = 5.30; *F*_(2, 675)_ = 0.098, *p* = 0.75], when age was controlled.

Table 1 shows the means and standard deviations for the variables. The scores in the EAT were high (*M* = 14.13, *SD* = 12.36) although they were below the cut-off point (*M* = 21) to identify the participants with possible eating disorders, Cyber victimization (ECIPQ) (*M* = 3.04, *SD* = 5.53), Appearance Evaluation (MBSRQ Subscale) (*M* = 3.43, *SD* =.85), Overweight Preoccupation (MBSRQ Subscale) (*M* = 2.48, *SD* = 0.96). Table 1 shows the zero-order correlations. Cyber victimization was positively correlated with the EAT and Overweight Preoccupation. Moreover, Cyber victimization was negatively correlated with Appearance Evaluation. Age only was correlated with the EAT. The rest of the correlations appear in Table 1.

**Table 1:** Correlations between Cyber victimization scores, Age, and psychopathology of eating disorders.

	***M* (*SD*)**	**1**	**2**	**3**	**4**	**5**
1. Cyber victimization	3.04 (5.53)	–	0.26^*^	−0.17^**^	0.19^**^	−0.01
2. Eating attitude test	14.13 (12.36)		–	−0.32^**^	0.58^**^	−0.14^**^
3. Appearance evaluation	3.43 (0.85)			–	−0.41^*^	0.06
4. Overweight preoccupation	2.48 (0.96)				–	−0.05
5. Age	14.28 (1.65)					–

* p < 0.05,

** *p < 0.01*.

**Figure 1:**
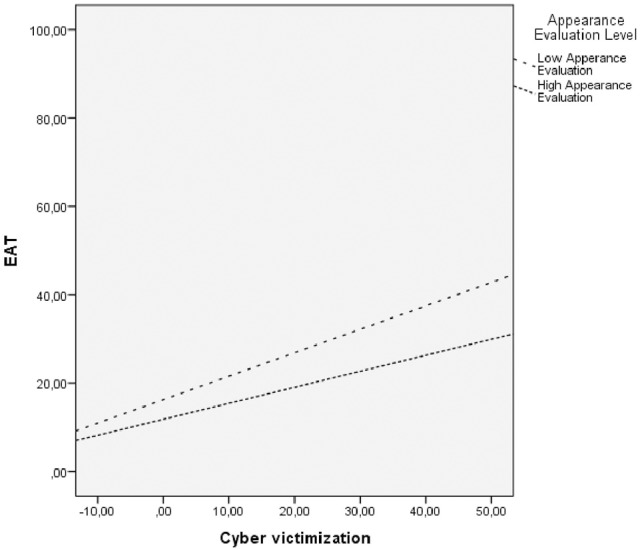
Appearance Evaluation moderates the association between Cyber victimization and eating disorder psychopathology. EAT, Eating Attitude Test.

As Table 2 shows, we can see that the model consisting of Gender, Age, Overweight Preoccupation, and Cyber victimization was a significant predictor of the EAT score [*R*^2^ = 0.40, *F*_(1, 650)_ = 45.02, *p* < 0.001]. The proposed model accounted for 40% of the variance in eating behaviors, attitudes, and symptoms associated with eating disorders. Once Gender, Age and Overweight Preoccupation had been entered, Cyber victimization was associated to eating behaviors, attitudes, and symptoms characteristic of eating disorders. When the Cyber victimization variable was added to the model, the percentage of explained variance increased significantly (Δ*R*^2^ = 0.04; *p* < 0.001). Values of standardized Beta coefficients of Cyber victimization were positive, so Cyber victimization was positively associated with eating behaviors, attitudes, and symptoms characteristic of eating disorders (EAT). Moreover, as Table 2 shows, Appearance Evaluation moderated the association between Cyber victimization and ED psychopathology (EAT) when Gender, Age, and Overweight Preoccupation were controlled. After Cyber victimization was entered, Appearance Evaluation predicted the eating disorder psychopathology (EAT), both in addition to Cyber victimization [*F*_(1, 649)_ = 4.44, *p* < 0.001] and when interacting with Cyber victimization, thus supporting a moderating impact of Appearance Evaluation in the association between Cyber victimization and the ED psychopathology (EAT) [*R*^2^ = 0.42, *F*_(1, 648)_ = 12.068, *p* < 0.01]. Figure 1 shows that in patients with higher levels of Appearance Evaluation increased Cyber victimization corresponded to smaller increases in the eating disorder psychopathology (EAT) than in the patients with low Appearance Evaluation. The Variance Inflation Factor (FIV) for the models were: Appearance Evaluation, FIV = 1.10; Overweight Preoccupation, FIV = 1.08, and Cyber victimization, FIV = 1.09; thus, these results suggest the absence of multicollinearity.

**Table 2:** Hierarchical regression analyses predicting eating disorders psychopathology (EAT) in adolescents.

**Step**	**Variable entered**	**Beta standardized**	**Total *R*^2^**	**Δ*R*^2^**
1	Gender	−0.041	0.36^**^	
	Age	−0.103^**^		
	Overweight preoccupation	0.535^**^		
2	Cyber victimization	0.151^**^	0.40^**^	0.04^**^
3	Appearance evaluation	−0.068^*^	0.41^**^	0.01^**^
4	Cyber Vic. X App. Eva.	−0.115^**^	0.42^**^	0.01^**^

Cyber Vic., Cyber Victimization. App. Eva., Appearance Evaluation.

* p < 0.05,

** *p < 0.001*.

## Discussion

Regarding our first aim, girls and boys had similar scores on Cyber victimization when Age was controlled. These results suggest that there are no statistically significant differences in Cyber victimization depending on the gender, agreeing with previous studies (e.g., Brown et al., 2014). Another main result of our study is that 57.5% of the adolescents have been victims of cyberbullying in the past 2 months. This percentage is greater than the frequency found in other studies, where the frequency ranged between 6.6% and 44.1% (Kowalski and Limber, 2007; Calvete et al., 2010; Garaigordobil, 2011; Hinduja and Patchin, 2013; Patchin and Hinduja, 2013; Rice et al., 2015). This discrepancy might be due to the fact that, in our study, the definition of cyberbullying was very broad and referred to a wide variety of cyberbullying behaviors in the past 2 months. Moreover, in other studies, cyber victimization was assessed with a single item and did not measure what types of electronic devices/media were used for the cyberbullying behavior (e.g., sms, Internet, social networks, and e-mail) or other behaviors (e.g., exclusion; Frisén et al., 2014; Arat, 2015; Ramos-Salazar, 2017). Therefore, several authors (e.g., Menzel et al., 2010) suggest that standardized questionnaires should be used to evaluate the different behaviors that can cause cyberbullying.

Regarding our second aim, the results showed that Cyber victimization was negatively correlated with Appearance Evaluation. This result agrees with previous studies that found that Cyber victimization was associated with body dissatisfaction in male and female adolescents (Frisén et al., 2014; Ramos-Salazar, 2017). These results can be explained by the fact that the body (shape, size and weight) is a main target of cyberbullying aggressors (Cassidy et al., 2009), and criticism, insults, and negative comments about the body are a fundamental aspect of the development of a negative body image in adolescents (Shroff and Thompson, 2006). Moreover, Cyber victimization was positively correlated with Overweight Preoccupation and with negative attitudes toward the food, calories intake, and eating behaviors associated with eating disorders, such as vomiting, binge-eating, or dieting.

Regarding the third aim, Cyber victimization was associated with eating disorder psychopathology, when age, gender and Overweight Preoccupation were controlled. Finally, Appearance Evaluation moderated the association between Cyber victimization and the ED psychopathology. All the variables had theoretical and empirical support justifying their inclusion in the analysis: Appearance Evaluation was associated with eating disorder psychopathology (e.g., Watson et al., 2011); Overweight Preoccupation is an important risk factor for eating disorders (Fairburn, 2008); and Cyber victimization was the main variable studied in our research. Thus, our results are consistent with another study that found Cyber victimization to be associated with the frequency of dieting to lose weight in adolescents (Ramos-Salazar, 2017). In addition, these results support other studies that found bullying and other forms of victimization are associated with body dissatisfaction, difficulties in regulating eating behavior, binge eating symptoms, and weight (e.g., Copeland et al., 2015). However, we would like to emphasize that this is the first study to indicate that Cyber victimization is associated with the psychopathology of eating disorders because we used an evaluation instrument that assesses the specific psychopathology of eating disorders.

These results support etiological theories of eating disorders such as the Tripartite Influence model of body image and eating disturbance (van den Berg et al., 2002). This theory suggests that three influences: peers, parents, and media have a direct effect on adolescents' body image dissatisfaction through two mediational processes: internalization of societal standards of appearance and excessive appearance comparison. In this regard, Cyber victimization is a consequence of the interaction with peers (Menzel et al., 2010) in social media (Tokunaga, 2010). Moreover, this model suggests that body dissatisfaction has a direct effect on restrictive eating and bulimic behaviors. This model has received previous support with adolescent samples (Shroff and Thompson, 2006).

The results of our study have practical implications: (a) It is necessary to assess the psychopathology of eating disorders (e.g., with EAT) in adolescents who have been victims of weight or appearance-based cyberbullying, and prevention programs for eating disorders should offer information about the negative consequences of cyberbullying to parents and educators. Finally, it is necessary to detect and evaluate cyberbullying in people diagnosed with eating disorders.

The present study has some limitations. First, we did not control the BMI of the participants, and body weight is one of the most common reasons that adolescents are bullied at school (Puhl et al., 2013). Likewise, we did not control the time spent daily on social media or the Internet, Machmutow et al. (2012) found that time spent online may increase the risk of cyber victimization. Therefore, these variables should be controlled in future studies. Moreover, we did not evaluate whether the participants were victims of cyberbullying focused on the body or weight. Considering that bullying due to the body and weight are the most frequent types in adolescents, future studies should evaluate the effect of cyber bullying directed specifically toward weight and the body, and its association with ED psychopathology. Furthermore, the percentage of variance explained when adding Cyber victimization to the model is low (4%), and the variance explained by the moderation model is very low (1%). However, these results are similar to those from other studies (e.g., Calvete et al., 2016; Ramos-Salazar, 2017), where the association between cyberbullying victimization and body dissatisfaction was weak (range of correlation from *r* = 0.10–0.13). Thus, it is possible that other mediating or moderating variables influence this association, for example, the participants' BMI.

Another limitation of this study is the fact that it is a cross-sectional study means that we cannot talk about causality between variables. Moreover, the study design was retrospective, and so the results obtained can be considered in terms of correlates rather than causal risk factors. Further research is needed to replicate this study using a longitudinal design. Moreover, our sample was composed of non-clinical participants, and future studies should replicate this research in participants with eating disorder diagnoses. Finally, Therefore, these limitations should always be taken into consideration when interpreting the results, which should be considered exploratory.

Technology is constantly evolving in a vast number of fields and cyber victimization is associated with psychopathology. Although this is a preliminary study, it is the first study to examine the association between Cyber victimization and eating disorder psychopathology in adolescents, and it indicates that Cyber victimization is associated with eating disorder psychopathology in adolescent girls and boys.

## Ethics statement

In this study the authors complied with APA ethical standards in the treatment of their participants and the research was approved by the University Ethics Committee of Catholic University of Valencia Sant Vincent Martir, Ref. UCV/2017-2018/60.

## Author contributions

JM designed the research, performed the statistical analyzes, and wrote the article. MT-I collected the sample and wrote the article.

### Conflict of interest statement

The authors declare that the research was conducted in the absence of any commercial or financial relationships that could be construed as a potential conflict of interest.

## References

[B1] Arat G. (2015). Emerging protective and risk factors of mental health in Asian American students: findings from the 2013 Youth Risk Behavior Survey. Vulnerable Child Youth Stud. 10, 192–205. 10.1080/17450128.2015.1045437

[B2] Beran T. Li Q. (2007). The relationship between cyberbullying and school bullying. J Stud Wellbeing 1, 15–33. 10.21913/JSW.v1i2.172

[B3] Brown C. F. Demaray M. K. Secord S. M. (2014). Cyber victimization in middle school and relations to social emotional outcomes. Comput. Human Behav. 35, 12–21. 10.1016/j.chb.2014.02.014

[B4] Calvete E. las Hayas C. del Barrio A. G. (2018). Longitudinal associations between resilience and quality of life in eating disorders. Psychiatry Res. 259, 470–475. 10.1016/j.psychres.2017.11.031 29149716

[B5] Calvete E. Orue I. Estévez A. Villardón L. Padilla P. (2010). Cyberbullying in adolescents: modalities and aggressors' profile. Comput. Human Behav. 26, 1128–1135. 10.1016/j.chb.2010.03.017

[B6] Calvete E. Orue I. Gamez-Guadix M. (2016). Cyberbullying victimization and depression in adolescents: the mediating role of body image and cognitive schemas in a one-year prospective study. Eur. J. Crim. Pol. Res. 22, 271–284. 10.1007/s10610-015-9292-8

[B7] Carmona-Torres J. A. Cangas A. J. Langer A. I. Aguilar-Parra J. M. Gallego J. (2015). Acoso escolar y su relación con el consumo de drogas y trastornos alimentarios: comparación entre adolescentes de Chile y España [Bullying and its relation to drug abuse and eating disorders: a comparison between Chile and Spain teenagers]. Psicol. Conduct. 23, 507–552.

[B8] Cash T. F. (2000). The MBSRQ Users' Manual, 3rd Edn. Norfolk, VA: Old Dominion University Available online at: www.body-images.com.

[B9] Cassidy W. Jackson M. Brown K. N. (2009). Sticks and stones can break my bones, but how can pixels hurt me? Students experiences with cyber-bullying. Sch. Psychol. Int. 30, 383–402. 10.1177/0143034309106948

[B10] Castro J. Toro J. Salamero M. Guimerá E. (1991). The Eating Attitudes Test: validation of the Spanish version. Psychol. Assess. 7, 175–190.

[B11] Cohen J. Cohen P. (1983). Applied Multiple Regression/Correlation Analysis for the Behavioral Sciences. Hillsdale, NJ: Lawrence Erlbaum.

[B12] Copeland W. E. Bulik C. M. Zucker N. Wolke D. Lereya S. T. Costello E. J. (2015). Does childhood bullying predict eating disorder symptoms? A prospective, longitudinal analysis. Int. J. Eat. Disord. 48, 1141–1149. 10.1002/eat.22459 26337405 PMC4715551

[B13] de Irala J. Cano-Prous A. Lahortiga-Ramos F. Gual-García P. Martínez-González M. A. Cervera-Enguix S. (2008). Validación del cuestionario Eating Attitudes Test (EAT) como prueba de cribado de trastornos de la conducta alimentaria en la población general. Med. Clin. 130, 487–491. 10.1157/13119489 18423166

[B14] Del Rey R. Casas J. A. Ortega-Ruiz R. Schultze-Krumbholz A. Scheithauer H. Smith P. (2015). Structural validation and cross-cultural robustness of the European Cyberbullying Intervention Project Questionnaire. Comput. Human Behav. 50, 141–147. 10.1016/j.chb.2015.03.065

[B15] Fairburn C. G. (2008). Cognitive Behavior Therapy and Eating Disorders. New York, NY: Guilford Press.

[B16] Frazier P. Tix A. P. Barron K. E. (2004). Testing moderator and mediator effects in counseling psychology research. J. Couns. Psychol. 51, 115–134. 10.1037/0022-0167.51.1.115

[B17] Frisén A. Berne S. Lunde C. (2014). Cybervictimization and body esteem: experiences of Swedish children and adolescents. Eur. J. Dev. Psychol. 11, 331–343. 10.1080/17405629.2013.825604

[B18] Garaigordobil M. (2011). Prevalencia y consecuencias del cyberbullying: una revisión. Rev. Int. Psicol. Ter. Psicol. 11, 233–254.

[B19] Garner D. M. Garfinkel P. E. (1979). The Eating Attitudes Test: an index of the symptoms of anorexia nervosa. Psychol. Med. 9, 273–279. 10.1017/S0033291700030762 472072

[B20] Hinduja S. Patchin J. W. (2008). Cyberbullying: an exploratory analysis of factors related to offending and victimization. Deviant. Behav. 29, 129–156. 10.1080/01639620701457816

[B21] Hinduja S. Patchin J. W. (2010). Bullying, cyberbullying, and suicide. Arch. Suicide Res. 14, 206–221. 10.1080/13811118.2010.494133 20658375

[B22] Hinduja S. Patchin J. W. (2013). Social influences on cyberbullying behaviors among middle and high school students. J. Youth Adolesc. 42, 711–722. 10.1007/s10964-012-9902-4 23296318

[B23] Kaltiala-Heino R. Rissanen M. Rimpela M. Rantanen P. (2003). Bulimia and impulsive behaviour in middle adolescence. Psychother. Psychosom. 72, 26–33. 10.1159/000067187 12466635

[B24] Kowalski R. M. Giumetti G. W. Schroeder A. N. Lattanner M. R. (2014). Bullying in the digital age: a critical review and meta-analysis of cyberbullying research among youth. Psychol. Bull. 140, 1073–1137. 10.1037/a0035618 24512111

[B25] Kowalski R. M. Limber S. P. (2007). Electronic bullying among middle school students. J. Adolesc. Health 41(Suppl. 6), S22–S30. 10.1016/j.jadohealth.2007.08.017 18047942

[B26] Lee K. Guy A. Dale J. Wolke D. (2017). Adolescent desire for cosmetic surgery: associations with bullying and psychological functioning. Plast. Reconstr. Surg. Glog. Open 139, 1109–1118. 10.1097/PRS.0000000000003252 28445361

[B27] Lunde C. Frisén A. Hwang C. P. (2007). Ten years old girls' body composition and peer victimization experiences: Prospective associations with body satisfaction. Body Image 4, 11–28. 10.1016/j.bodyim.2006.10.002 18089248

[B28] Machmutow K. Perren S. Sticca F. Alsaker F. D. (2012). Peer victimisation and depressive symptoms: can specific coping strategies buffer the negative impact of cybervictimisation? Emot. Behav. Difficul. 17, 403–420. 10.1080/13632752.2012.704310

[B29] Marco J. H. Perpiñá C. Roncero M. Botella C. (2017). Confirmatory factor analysis and psychometric properties of the Spanish version of the Multidimensional Body-Self Relations Questionnaire-Appearance Scales in early adolescents. Body Image 21, 15–18. 10.1016/j.bodyim.2017.01.003 28229919

[B30] Menzel J. E. Schaefer L. M. Burke N. L. Mayhew L. L. Brannick M. T. Thompson J. K. (2010). Appearance-related teasing, body dissatisfaction, and disordered eating: a meta-analysis. Body Image 7, 261–270. 10.1016/j.bodyim.2010.05.004 20655287

[B31] Mishna F. Cook C. Gadalla T. Daciuk J. Solomon S. (2010). Cyber bullying behav-iors among middle and high school students. Am. J. Orthopsychiatry 80, 362–374. 10.1111/j.1939-0025.2010.01040.x 20636942

[B32] Patchin J. W. Hinduja S. (2013). Cyberbullying among adolescents: implications for empirical research. J. Adolesc. Health 53, 431–432. 10.1016/j.jadohealth.2013.07.030 24054078

[B33] Pennesi J. L. Wade T. D. (2016). A systematic review of the existing models of disordered eating: do they inform the development of effective interventions? Clin. Psychol. Rev. 43, 175–192. 10.1016/j.cpr.2015.12.004 26781985

[B34] Puhl R. M. Peterson J. L. Luedicke J. (2013). Weight-based victimization: Bullying experiences of weight loss treatment–seeking youth. Pediatrics 131, e1–e9. 10.1542/peds.2012-1106 23266918

[B35] Ramos-Salazar L. (2017). Cyberbullying victimization as a predictor of cyberbullying perpetration, body image dissatisfaction, healthy eating and dieting behaviors, and life satisfaction. J. Interpers. Violence. [Epub ahead of print]. 10.1177/0886260517725737 29294894

[B36] Raskauskas J. Stoltz A. D. (2007). Involvement in traditional and electronic bullying among adolescents. Develop. Psychol. 43, 564–575. 10.1037/0012-1649.43.3.564 17484571

[B37] Rice E. Petering R. Rhoades H. Winetrobe H. Goldbach J. Plant A.. (2015). Cyberbullying perpetration and victimization among middle-schoolstudents. Am. J. Public Healt. 105, e66–e72. 10.2105/AJPH.2014.302393 25602905 PMC4330864

[B38] Selkie E. M. Kota R. Chan Y.-F. Moreno M. (2015). Cyberbullying, depression, and problem alcohol use in female college students: a multisite study. Cyberpsychol. Behav. Soc. Netw. 18, 79–86. 10.1089/cyber.2014.0371 25684608 PMC4323024

[B39] Shroff H. Thompson J. K. (2006). The tripartite influence model of body image and eating disturbance: a replication with adolescent girls. Body Image 3, 17–23. 10.1016/j.bodyim.2005.10.004 18089205

[B40] Sweetingham R. Waller G. (2008). Childhood experiences of being bullied and teased in the eating disorders. Eur. Eat. Disord. Rev. 16, 401–407. 10.1002/erv.839 17960780

[B41] Tokunaga R. S. (2010). Following you home from school: a critical review and synthesis of research on cyberbullying victimization. Comput. Human Behav. 26, 277–287. 10.1016/j.chb.2009.11.014

[B42] van den Berg P. Thompson J. K. Obremski-Brandon K. Coovert M. (2002). The Tripartite Influence model of body image and eating disturbance. J. Psychos. Res. 53, 1007–1020. 10.1016/S0022-3999(02)00499-3 12445590

[B43] Watson H. J. Raykos B. C. Street H. Fursland A. Nathan P. R. (2011). Mediators between perfectionism and eating disorder psychopathology: Shape and weight overvaluation and conditional goal-setting. Int. J. Eat. Disord. 44, 142–149. 10.1002/eat.20788 20127937

[B44] World Health Organization and UNICEF. (2003). Global Strategy for Infant and Young Child Feeding. World Health Organization.

[B45] Ybarra M. L. Mitchell K. J. (2004). Online aggressor/targets, aggressors, and targets: a comparison of associated youth characteristics. J. Child Psychol. Psychiatry 45, 1308–1316. 10.1111/j.1469-7610.2004.00328.x 15335350

